# Knowledge as resistance: advancing global health in challenging times

**DOI:** 10.1002/jia2.26430

**Published:** 2025-02-26

**Authors:** Marlène Bras, the Editors of the Journal of the International AIDS Society

**Affiliations:** ^1^ Journal of the International AIDS Society

These are extraordinary times, with daily assaults on public health principles. The sudden de‐funding of life‐saving programmes like PEPFAR poses existential threats to achieving the goals of effective control of the HIV epidemic, which had been predicated on increasing testing, engagement in care, and uptake of evidence‐based treatment and prevention. The censorship of terms related to sex, gender, diversity, equity, and inclusion in public health reports and scientific publications is chilling and negates decades of thoughtful scholarship that has demonstrated the relevance of these issues in the lived experiences and health outcomes of people affected by HIV.

The leadership of IAS – the International AIDS Society – has expressed deep concern over the rhetoric surrounding funding cuts, which has misrepresented the use of international development support and needlessly further stigmatized vulnerable populations. As editors of the *Journal of the International AIDS Society*, we affirm that the journal will not change our mission nor our standards for evidence‐driven and person‐centred reporting. We recognize that programmes funded with public money should be subject to government review. However, the acute pause in US funding for global health, along with statements and policy shifts targeting key populations, represent unprecedented attacks on initiatives that have saved millions of lives and prevented millions of new HIV acquisitions.

JIAS will continue to welcome research that is of interest to our diverse readership, can inform the development of effective strategies to decrease HIV transmission, and improve the health and wellbeing of people living with HIV across the globe. Our core principles continue to include an understanding that sex and gender are distinct, and that rigorous science respects the diversity of human experience.

As researchers, clinicians and public health practitioners, we must respond to the challenges posed by recent policy measures while also documenting their intended and unintended consequences. JIAS welcomes submissions that present empirical data, model potential outcomes of resource constraints and policy shifts or highlight best practices and innovative solutions that address current challenges.

We have received requests from authors of manuscripts under peer review to have their names removed or manuscripts withdrawn from further consideration in order to comply with the US administration's recent executive orders. Although we are very concerned about this, we will respect author requests in agreement with our policy on authorship changes.

This is a difficult time, but the global struggle to address the HIV epidemic has faced and overcome existential challenges before. We feel that the best way to counter misinformation is to continue to generate and disseminate new knowledge that provides a compelling, evidence‐based narrative. While an instinctive response to such an unprecedented threat to hard‐earned progress may be to retreat, we believe that the opposite approach is needed in order to support people and global health equity. It is essential to continue research that strengthens the case for effective strategies to address HIV and related epidemics.

In the face of uncertainty and adversity, our collective commitment to rigorous science, inclusivity and truth must remain unwavering.

## COMPETING INTERESTS

The authors declare no competing interests.

## AUTHORS’ CONTRIBUTIONS

The Editors‐in‐Chief and the Executive Editor have collectively contributed to the writing and revision of the manuscript. All have approved the final manuscript.

## DISCLAIMER

The views in this article are those of the authors in their capacity of Editors, and do not necessarily represent the views, decisions or policies of the institutions with which they are affiliated.

